# Women with short survival after diagnosis of metastatic breast cancer: a population-based registry study

**DOI:** 10.1007/s10549-022-06591-7

**Published:** 2022-04-24

**Authors:** Caroline Boman, Luisa Edman Kessler, Jonas Bergh, Alexios Matikas, Theodoros Foukakis

**Affiliations:** 1 Department of Oncology and Pathology, Karolinska Institute, Visionsgatan 4, Bioclinicum, 171 74 Stockholm, Sweden; 2 Breast Center, Theme Cancer, Karolinska University Hospital, Stockholm, Sweden

**Keywords:** Breast cancer, Metastatic, Population, Prognosis, Short survival

## Abstract

**Purpose:**

Despite therapeutic advances, overall survival of metastatic breast cancer (MBC) at the population level has seen little improvement over the past decades. Aggressive tumor biology or delay in access to cancer care might be contributing factors. With this retrospective population-based study we aimed to quantify and characterize patients with very short survival time following MBC diagnosis.

**Methods:**

Women diagnosed with MBC between Jan 1st, 2005 and Dec 31st, 2016 were identified using the population-based Stockholm–Gotland breast cancer registry. Data regarding demographic and clinicopathological characteristics, survival, and treatment were extracted retrospectively from the registry and from patient charts. Patients who died within 90 days following diagnosis of MBC were identified and their characteristics were compared with all other patients diagnosed with MBC during the same period.

**Results:**

Between 2005 and 2016, 3124 patients were diagnosed with MBC, of whom 498 (16.2%) died within 90 days of diagnosis. Nearly half (*N* = 233) did not receive any antitumoral treatment. Patients with short survival were older (*p* < 0.001), had higher primary tumor grade (*p* < 0.001), higher clinical stage at primary diagnosis (*p* = 0.002), and more often estrogen receptor (ER)-negative breast cancer (*p* < 0.001). Visceral metastases were more frequent (*p* < 0.001) and patients with short survival received adjuvant chemotherapy (*p* < 0.001) to a lesser extent compared to patients with a better prognosis. In multivariable analysis older age, time period of diagnosis, metastasis site, adjuvant chemotherapy, and primary tumor grade were independent predictors for short survival, whereas ER status was not.

**Conclusion:**

Nearly one out of six patients with MBC survive less than 3 months after diagnosis. Our findings demonstrate a different spectrum of MBC at population level and can potentially inform on individualized follow-up strategies and treatment algorithms.

**Supplementary Information:**

The online version contains supplementary material available at 10.1007/s10549-022-06591-7.

## Introduction

Breast cancer (BC) is the most common malignancy in women globally, with more than 2.2 million new cases annually. Accounting for more than 15% of all cancer deaths among women, breast cancer comprises the leading cause of female cancer mortality worldwide [1, 2]. Metastatic BC (MBC) is with few exceptions considered an incurable disease where treatment is given to palliate symptoms and to prolong life, until disease progression or unacceptable toxicity occurs [3–6].

Despite therapeutic advances and significant improvements in prognosis according to controlled clinical trials [7–12], the improvement of overall survival of MBC at population level is less pronounced [13]. For example, in a resource-rich setting, as is the case in the Stockholm–Gotland region in Sweden, where screening, treatment, and surveillance are available to all women irrespective of socioeconomic status and health insurance, overall survival (OS) has stagnated over the past decades [14, 15]. Accordingly, in a recent meta-analysis 12 out of 15 studies showed improvement in unadjusted survival over time; however, in only 3 of 10 studies the improvement remained significant at multivariate analysis adjusting for potential confounding variables [16].

Patients with metastatic malignancies included in prospective randomized trials are often more fit, with fewer comorbidities and younger age compared to the general patient population [17–21]. However, differences in comorbidities may not entirely explain the discrepancy in prognosis. One potential contributing factor is women with MBC with very short survival after diagnosis, a population de facto excluded from controlled trials. This short survival, which might be attributed to the biology of the disease or delay in access to cancer care, implies that at least some patients never receive any treatment. Knowledge regarding these women with extremely poor prognosis is scarce, with a lack of studies shedding light on this population. Herein we aim to provide real-world data to better understand the incidence and characteristics of women with short survival after MBC diagnosis.

## Materials and methods

### Study design

This is a retrospective, ad hoc cohort study whose primary aim was to characterize patients with short survival after MBC diagnosis. The secondary aim was to perform a comparison with the general population of MBC patients in the same region in order to identify possible differences and potential prognostic factors. Short survival was defined, for the scope of this study, as death within 90 days of MBC diagnosis. Patients included in the study were identified through the Stockholm–Gotland breast cancer registry (SBCR), as described below. The study was approved by the ethics committee in Stockholm (decision number 2016/1303/31 and 2018/642-32).

### Patients and data collection

Women aged 18 years and above, diagnosed with MBC during the period of January 1st, 2005 to December 31st, 2016, were identified through the SBCR. This is a population-based registry in which cases of BC diagnosed in the Stockholm–Gotland area, Sweden have been reported since 1976. It contains both demographic and clinicopathological data and is a part of the national breast cancer registry which has been shown to have excellent cover [22]. Cases of relapse/metastasis are reported by the clinician and/or pathologist. In addition, identification of non-reported cases is done yearly in the registry by manually checking the presence of metastatic diagnosis in all patients with breast cancer as cause of death in the Swedish death registry. The date of diagnosis was defined as the time of diagnosis of metastatic disease, verified either through histology, cytology, or imaging, whichever came first. For cutaneous metastases, the date of the clinical diagnosis by the physician was accepted.

For the entire population of patients diagnosed with MBC during this time period, data were collected from the SBCR regarding date of primary diagnosis, date of metastatic disease diagnosis, date of death, metastatic site, treatment of primary tumor, stage at time of primary diagnosis, as well as data on primary tumor characteristics, such as Nottingham Histologic Grade (NHG), estrogen receptor (ER), progesterone receptor (PR), and Human Epidermal Growth Factor Receptor 2 (HER2) expression. Women who died within 90 days of MBC diagnosis were eligible for the study cohort, with the rest comprising the control cohort. For the patients in the study cohort, additional data were manually collected from medical records using electronic hospital charts. Information was collected regarding pathological characteristics of the primary tumor and metastases, number of metastatic sites, as well as data on comorbidities and treatment in the metastatic setting. In addition, the medical records were used to verify that the date of first MBC diagnosis was correct and that the metastatic disease was indeed due to BC and not another primary cancer.

Women with bilateral BC contributed with pathological characteristics from only one tumor. For metachronous BC—here defined as tumors diagnosed more than 90 days apart—data on the latest tumor were included. For synchronous BC, the tumor assessed to have the most data on pathological characteristics, also being the most clinically relevant, was included. De novo metastatic disease was defined as less than 90 days between primary diagnosis and diagnosis of metastatic disease. Short distant recurrence-free interval (DRFI) was defined as less than 48 months between primary diagnosis and diagnosis of metastatic disease. MBC was defined as disease with distant metastasis, excluding locoregional lymph node metastasis.

Exclusion criteria from the study cohort were histology or cytology showing other metastatic cancer besides BC, concurrent metastatic cancer in addition to MBC and death occurring more than 90 days from MBC diagnosis. End of follow-up was set to Dec 31st, 2017.

### Statistical analysis

As the main aim of this study was descriptive, no formal statistical hypothesis was tested. Frequency tables were used to summarize categorical variables along with descriptive statistics used for continuous variables. For comparison of categorical variables, the Pearson’s Chi-squared test was used. For comparison of paired categorical variables (receptor expression at primary disease versus metastasis), McNemar’s test was used. Overall survival was measured from the date of MBC diagnosis until the date of death from any cause or the date of last follow-up. DRFI was measured from the date of primary BC diagnosis to MBC diagnosis. Logistic regression was used to identify factors associated with short survival after diagnosis of MBC. Cochran–Armitage chi-square test as well as linear-by-linear association test was performed to identify time trends in survival. The statistically significant level was set to 5% (two-tailed). The statistical analysis was performed using IBM SPSS Statistics version 25 (IBM, NY, USA).

## Results

### Description of study cohort and patient characteristics

**Fig. 1:**
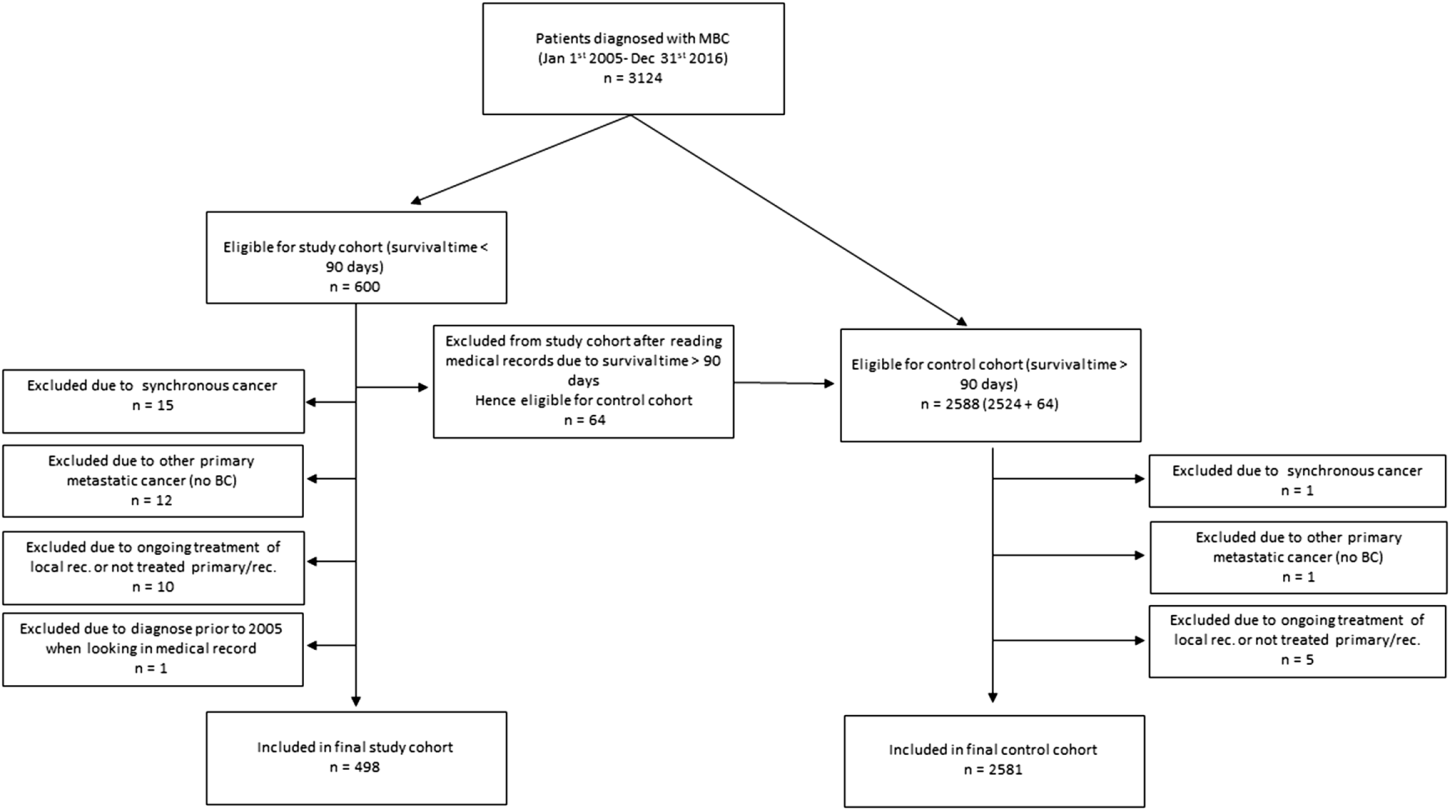
Flowchart of patient selection and inclusion to study and control cohorts

Between Jan 1st, 2005 and Dec 31st, 2016, a total of 3124 women were diagnosed with MBC in the Stockholm–Gotland region, of whom 600 women were registered to have an OS of 90 days or less, thus being eligible for inclusion in the study cohort. After reviewing medical records, 102 women were excluded from the study cohort; 64 due to discrepancy in registered OS, 15 due to other active synchronous cancers, 12 due to metastasis from another primary cancer, 10 due to ongoing treatment for local recurrence or having untreated primary BC, and one due to diagnosis prior to Jan 1st, 2005. As a result, 498 women were enrolled in the final study cohort. From the control cohort, 7 patients were excluded, leaving 2581 women in the final control cohort (Fig. 1). Thus, almost one out of six women (16.2%) died within 90 days of MBC diagnosis.

The demographic and clinicopathological characteristics of the patients included in the study are summarized in Table 1. There was a significant age difference between the two groups, with a median age of 73.4 years (26.5–97.6) in the study cohort vs. 65.9 years (21.8–99.5) in the control cohort (median test *p* < 0.001). Patients in the study population had a higher primary disease grade compared to the control cohort (Pearson’s chi-square *p* = 0.001), more advanced clinical stage (Pearson’s chi-square *p* = 0.002), and less often hormone receptor-positive disease, with 32.7% being ER-negative in the primary setting as opposed to 18.2% in the control group (Pearson’s chi-square *p* < 0.001). This difference was also seen regarding PR status on primary tumor, with 49.0% being PR-negative in the study group vs. 31.4% in the control group (Pearson’s chi-square p < 0.001). No difference regarding HER2 status was detected; however, there was considerable missingness regarding HER2 data. There was no significant difference in de novo metastatic disease between the study and control cohort (16.7% versus 17.4%, Pearson’s chi-square *p* = 0.693). In the study cohort, 23.2% of patients for whom receptor status was available for both the primary tumor and the metastasis showed ER conversion (McNemar’s test *p* = 0.026), 33.7% had PR conversion (McNemar’s test *p* < 0.001), and 10.1% had HER2 conversion (McNemar’s test *p* = 1.000) (supplementary table 1).

**Table 1:** Demographic and clinicopathological characteristics of patients diagnosed with metastatic breast cancer in Stockholm–Gotland region 2005–2016

	Study cohort	Control cohort	*p* value
	*N* = 498 (%)	*N* = 2581 (%)	
Age			
Median (min–max)	73.4 (26.5–97.6)	65.9 (21.8–99.5)	< 0.001
De novo metastatic disease			
Yes	83 (16.7)	449 (17.4)	0.693
No	415 (83.3)	2132 (82.6)	
Site of metastasis by group			
CNS/visceral	283 (56.8%)	952 (36.9%)	< 0.001
Bone/nodal/skin only	86 (17.3%)	976 (37.8%)	
Other/unknown	129 (25.9%)	653 (25.3%)	
Estrogen receptor primary			
Positive	296 (59.4)	1585 (61.4)	< 0.001
Negative	163 (32.7)	471 (18.2)	
Not available	39 (7.8)	525 (20.3)	
Progesterone receptor primary			
Positive	205 (41.2)	1218 (47.2)	< 0.001
Negative	244 (49.0)	810 (31.4)	
Not available	49 (9.8)	553 (21.4)	
HER2 primary			
Positive	43 (8.6)	160 (6.2)	0.640
Negative	188 (37.8)	631 (24.4)	
Not available	267 (53.6)	1790 (69.4)	
Primary disease grade			
I	5 (1.0%)	61 (2.4%)	< 0.001
II	93 (18.7%)	534 (20.7%)	
III	187 (37.6%)	587 (22.7%)	
Unknown	213 (42.8%)	1399 (54.2%)	
Clinical stage at primary diagnosis			
I	104 (20.9%)	753 (29.1%)	0.002
II	248 (49.8%)	1127 (43.7%)	
III	54 (10.8%)	212 (8.2%)	
IV	78 (15.7%)	426 (16.5%)	
Unknown	14 (2.8%)	64 (2.5%)	
Adjuvant chemotherapy			
Yes	156 (31.3%)	952 (36.9%)	< 0.001
No	91 (18.3%)	328 (12.7%)	
Unknown	251 (50.4%)	1301 (50.4%)	

The most common site of metastasis in patients with short survival was CNS or viscera as opposed to bone, lymph node, or skin, which were the most common site of metastasis in all other patients (Pearson’s chi-square *p* < 0.001).

### Treatment patterns in the metastatic setting

Of the 498 women included in the study cohort only 47.4% (*n* = 236) received antitumoral therapy. Systemic treatment was given to 37.8% of the women, endocrine treatment (ET) being most common (19.9%), followed by chemotherapy (17.7%). In addition, 9.6% of the included women received radiotherapy only, whereas no treatment information was available for 29 women (5.8%).

As a result, 233 women (46.8%) in the study cohort did not receive any antitumoral therapy. For 189 (81.1%) of these, no treatment recommendation was made. This was due to death during the initial work-up (*n* = 41), liver failure (*n* = 37), or poor performance status (PS)/comorbidities (*n* = 93). In 15 cases, no treatment recommendation was given due to patient demand. In 3 cases, the reason for a lack of treatment recommendation was unknown. Moreover, for 33 women (14.1%) recommendation was made but no treatment was administered due to death prior to treatment initiation (*n* = 23), poor PS (*n* = 6), or patient demand (n = 3). In one case, the reason was unknown. For 11 women, no information on treatment recommendation was found.

Women in the study cohort that received treatment were significantly younger than those who did not (median test *p* < 0.001). The presence of liver metastases was negatively associated with treatment (Pearson’s chi-square *p* = 0.008), whereas there was a positive association with the presence of CNS metastases (Pearson’s chi-square *p* < 0.001), due to a high proportion of women receiving whole-brain irradiation. There were no differences in treatment patterns according to ER status of metastatic disease (Pearson’s chi-square *p* = 0.307), HER2 expression at metastatic disease (Pearson’s chi-square *p* = 0.077), or DRFI (Pearson’s chi-square *p* = 0.658). However, a temporal trend toward fewer patients receiving cancer therapy in recent years was found, from 55.3% during 2005–2008 to 42.2% during 2013–2016 (Cochran–Armitage test for trend *p* = 0.018). This trend was significant for radiotherapy (Cochran–Armitage test for trend *p* < 0.001) but not chemotherapy (Cochran–Armitage test for trend *p* = 0.812) or endocrine therapy (Cochran–Armitage test for trend *p* = 0.773).

### Predictors of short survival

**Fig. 2:**
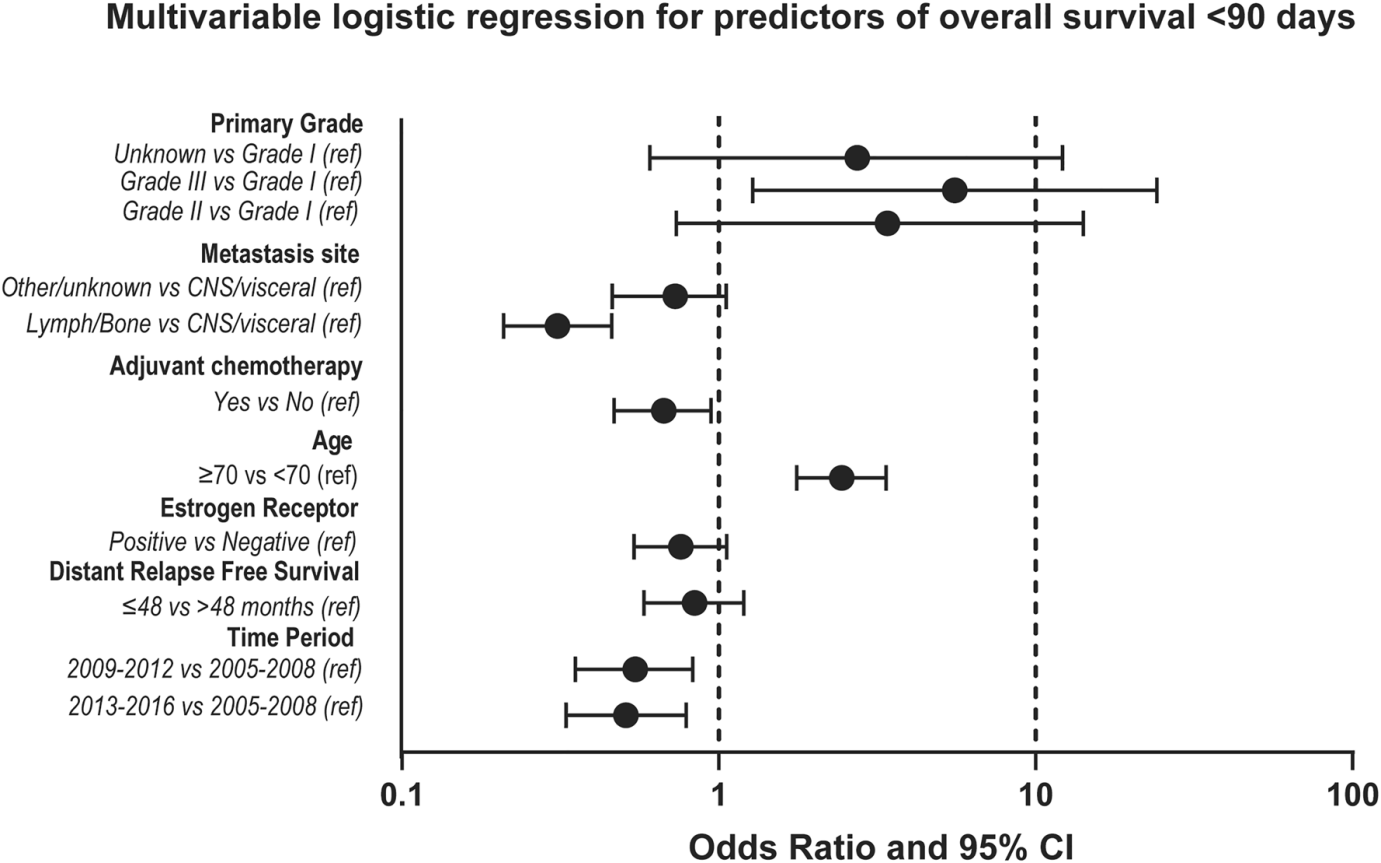
Multivariable logistic regression for predictors of overall survival < 90 days from metastatic breast cancer diagnosis

The results of univariate and multivariable logistic regressions in respect to inclusion to the study cohort are presented in Table 2. All predictors of short survival in univariate analysis were included to the multivariable model. In multivariable analysis, five factors remained significantly associated with short survival: age, time period, metastasis site, adjuvant chemotherapy, and primary tumor grade (Fig. 2, Table 2).

**Table 2:** Binary logistic regression for predictors of survival < 90 days

	Univariate OR (95% CI)	*p* value	Multivariable OR (95% CI)	*p* value
Age		< 0.001		< 0.001
≤ 70	1 (reference)		1 (reference)	
> 70	2.30 (1.89–2.80)		2.44 (1.76–3.37)	
ER status at primary		< 0.001		0.112
Positive	0.54 (0.43–0.67)		0.76 (0.54–1.06)	
Negative	1 (reference)		1 (reference)	
DRFI		< 0.001		0.343
≤ 48 months	1 (reference)		1 (reference)	
> 48 months	0.54 (0.44–0.67)		0.84 (0.58–1.20)	
Time Period		0.026		0.005
2005–2008	1 (reference)		1 (reference)	
2009–2012	0.73 (0.58–0.92)		0.54 (0.35–0.82)	
2013–2016	0.81 (0.64–1.02)		0.51 (0.33–0.79)	
Metastasis site		< 0.001		< 0.001
CNS/visceral	1 (reference)		1 (reference)	
Lymph/bone	0.29 (0.23–0.38)		0.31 (0.21–0.46)	
Other/unknown	0.66 (0.52–0.83)		0.73 (0.48–1.10)	
Adjuvant chemo		< 0.001		0.036
Yes	0.59 (0.44–0.78)		0.69 (0.48–0.97)	
No	1 (reference)		1 (reference)	
Primary grade		< 0.001		0.001
I	1 (reference)		1 (reference)	
II	2.12 (0.83–5.42)		3.21 (0.73–14.03)	
III	3.88 (1.54–9.81)		5.56 (1.28–24.15)	
Unknown	1.85 (0.73–4.67)		2.74 (0.61–12.23)

## Discussion

While the issues of MBC survivorship and potential factors associated with it have been the focus of many studies, this is to our knowledge the first study to solely focus on MBC patients with very short survival time following diagnosis. In this retrospective cohort study, we show that almost one out of six women diagnosed with MBC had an extremely poor prognosis, with an overall survival of 90 days or less from time of diagnosis of metastatic disease. This fact illustrates the importance to identify and characterize this relatively large patient population in order to ensure adequate and timely diagnostic and therapeutic interventions.

Multiple factors may have contributed to the observed short survival. The median age among women in the study cohort was significantly higher than in the control cohort, which is in line with previous studies showing that age is a prognostic factor in women with BC [23–25]. In addition, older women more often present with comorbidity and comorbidity alone have been associated with a worse prognosis both in early and advanced BC, potentially constituting an additional contributing factor [26–28]. Furthermore, older women are more frequently subject to undertreatment and may therefore present with a worse prognosis further explaining some of the age difference seen in this study [29, 30]. Beyond demographic factors, tumor biology is a decisive factor that determines MBC prognosis. This was illustrated by the overrepresentation of higher tumor grade, receptor conversion to ER-negative metastasis from an ER-positive primary, and higher clinical stage at primary diagnosis as well as a higher frequency of visceral metastases seen in the study cohort, all known negative prognostic factors [24, 31–35]. Adjuvant chemotherapy was more common among women in the control cohort and was also shown to be a positive predictor for survival after MBC diagnosis. However, due to considerable missingness, these data should be interpreted with caution. Taken together, the presence of a higher stage at primary diagnosis and less adjuvant chemotherapy may indicate that these patients have been subject to undertreatment.

Remarkably, nearly half of the study cohort did not receive any antitumoral treatment, an observation previously described in another patient cohort [36]. As a result, these women did not get the opportunity of the potential benefits of modern therapy. Delay in seeking health care services may result in patients presenting in late stages of disease, thus being disqualified for active treatment due to poor PS and comorbidities. On the other hand, delays in diagnostic procedures or referral to oncologic care, as well as prolonged time to treatment initiation may also be possible contributing factors. Patient refusal of antitumoral therapy has been reported to be more common among critically ill and elderly patients [37, 38]. In this study, however, only 18 patients (7%) clearly declined treatment. Among untreated women, no treatment recommendation was made in most cases, often due to poor PS or death occurring before confirmation of diagnosis or treatment initiation, which further illustrates the need for swift handling and care of patients with advanced MBC. Interestingly, time period of MBC diagnosis was shown to be a significant predictor of survival with a decreasing proportion of women with short survival over time, which may reflect improvements in cancer treatment. Nevertheless, a trend showing fewer patients receiving treatment among those with short survival was noted.

An advantage of this study and one of the reasons for our findings is that the women included were identified from a population-based registry that includes all women diagnosed with MBC regardless of whether they are referred to an oncology service or not. In addition, all women with BC as cause of death without registered MBC diagnosis are retrospectively checked and any absent registrations are corrected. This results in a registry with good coverage, providing a representative sample of the whole-MBC population.

The study also has its limitations. As in all retrospective cohort studies, selection bias cannot be excluded. There was considerable missingness regarding registry data on tumor characteristics, including receptor status from primary diagnosis, especially HER2 data, which may affect the interpretation of the results. As for missingness regarding tumor characteristics in the metastatic setting, there are several plausible explanations. In some cases, treatment may have been given on vital indication before the diagnostic work-up, including prior to completion of biopsy for tumor characteristics. Work-up may also be discontinued due to patients’ deteriorating PS. In addition, some tumors, such as CNS metastasis, may not be available for biopsy. Another limitation of this study is the lack of knowledge with regards to treatment of MBC in the control cohort, making a direct comparison between the two cohorts impossible.

In summary, this study provides real-world evidence on survival after MBC diagnosis, demonstrating that one out of six women had a poor prognosis with survival shorter than 90 days from time of diagnosis. A large proportion of these women never had the chance to receive and potentially benefit from modern therapy. We propose that early identification of these women may impact the stagnation of OS seen at the population level.

## Supplementary Information

Below is the link to the electronic supplementary material.

Supplementary file1 (DOCX 17 KB)

## Data Availability

The data that support the findings of this study are available on reasonable request from the corresponding author [CB].
