# Supplementary material for: Women with short survival after diagnosis of metastatic breast cancer: a population-based registry study
Source: Breast Cancer Res Treat. 2022 Apr 24;194(1):49–56. doi: 10.1007/s10549-022-06591-7 (PMC9167164; doi:10.1007/s10549-022-06591-7)
Supplement: Supplementary file 1 — Supplementary file1 (DOCX 17 KB) [file 10549_2022_6591_MOESM1_ESM.docx]

**Supplementary material**

**Supplementary table 1.** Receptor conversion primary vs local relapse vs metastasis (study group).

| **Primary vs metastasis** | | | | | |
| --- | --- | --- | --- | --- | --- |
|  | **Both positive** | **Both negative** | **(+) → (-)** | **(-) → (+)** | **P** |
| **ER** | 85 | 48 | 27 | 13 | 0.038 |
| **PR** | 22 | 83 | 51 | 4 | <0.001 |
| **HER2** | 7 | 47 | 3 | 3 | 1.000 |
|  |  |  |  |  |  |
| **Primary vs metastasis or local relapse** | | | | | |
| **ER** | 92 | 60 | 31 | 15 | 0.026 |
| **PR** | 24 | 98 | 58 | 4 | <0.001 |
| **HER2** | 9 | 53 | 4 | 3 | 1.000 |
|  |  |  |  |  |  |

P-values by McNemar’s test. More patients converted from estrogen/progesterone receptor positive primary tumor to hormone receptor negative metastasis, than vice versa. HER2 expression was stable from primary to metastatic tissue.

**Supplementary table 2.** Site of metastasis in women with short survival after metastatic breast cancer diagnosis (study group).

| **Site of metastasis** | **n = 498 (%)** |
| --- | --- |
| CNS | 73 (14.7) |
| Liver | 256 (51.4) |
| Bone | 246 (49.4) |
| Lung | 193 (38.8) |
| Lymph nodes | 88 (17.7) |
| Locally advanced | 8 (1.6) |
| Skin | 46 (9.2) |
| Other sites | 80 (16.1) |
